# Persistence of anxiety symptoms after elective caesarean delivery

**DOI:** 10.1192/bjo.2018.48

**Published:** 2018-08-17

**Authors:** Anna B. Janssen, Katrina A. Savory, Samantha M. Garay, Lorna Sumption, William Watkins, Isabel Garcia-Martin, Nicola A. Savory, Anouk Ridgway, Anthony R. Isles, Richard Penketh, Ian R. Jones, Rosalind M. John

**Affiliations:** Research Associate, Biomedicine Division, School of Biosciences, Cardiff University, UK; Research Assistant, Biomedicine Division, School of Biosciences, Cardiff University, UK; PhD student, Biomedicine Division, School of Biosciences, Cardiff University, UK; Statistician, Infection and Immunity Team Bioinformatics and Statistics, College of Biomedical & Life Sciences, Cardiff University, UK; Research Midwife, Department of Obstetrics and Gynaecology, University Hospital Wales, UK; Professor, National Centre for Mental Health, MRC Centre for Neuropsychiatric Genetics and Genomics, School of Medicine, Cardiff University, UK; Consultant Obstetrician and Gynaecologist, Department of Obstetrics and Gynaecology, University Hospital Wales, UK; Professor, National Centre for Mental Health, MRC Centre for Neuropsychiatric Genetics and Genomics, School of Medicine, Cardiff University, UK; Professor, Biomedicine Division, School of Biosciences, Cardiff University, UK

**Keywords:** Perinatal psychiatry, anxiety disorders, elective caesarean

## Abstract

**Background:**

In the UK, 11.8% of expectant mothers undergo an elective caesarean section (ELCS) representing 92 000 births per annum. It is not known to what extent this procedure has an impact on mental well-being in the longer term.

**Aims:**

To determine the prevalence and postpartum progression of anxiety and depression symptoms in women undergoing ELCS in Wales.

**Method:**

Prevalence of depression and anxiety were determined in women at University Hospital Wales (2015–16; *n* = 308) through completion of the Edinburgh Postnatal Depression Scale (EPDS; ≥13) and State-Trait Anxiety Inventory (STAI; ≥40) questionnaires 1 day prior to ELCS, and three postpartum time points for 1 year. Maternal characteristics were determined from questionnaires and, where possible, confirmed from National Health Service maternity records.

**Results:**

Using these criteria the prevalence of reported depression symptoms was 14.3% (95% CI 10.9–18.3) 1 day prior to ELCS, 8.0% (95% CI 4.2–12.5) within 1 week, 8.7% (95% CI 4.2–13.8) at 10 weeks and 12.4% (95% CI 6.4–18.4) 1 year postpartum. Prevalence of reported anxiety symptoms was 27.3% (95% CI 22.5–32.4), 21.7% (95% CI 15.8–28.0), 25.3% (95% CI 18.5–32.7) and 35.1% (95% CI 26.3–44.2) at these same stages. Prenatal anxiety was not resolved after ELCS more than 1 year after delivery.

**Conclusions:**

Women undergoing ELCS experience prolonged anxiety postpartum that merits focused clinical attention.

**Declaration of interest:**

None.

There is an impact in one in four pregnancies from stress, depression and/or anxiety with the risk of these conditions highest in women with a history of mental illness and those exposed to adverse circumstances.^1^^–^^3^ Antenatal depression is highly comorbid with antenatal anxiety^4^^,^^5^ and both are risk factors for postpartum depression that can impair mother–infant interactions.^6^ Antenatal depression and anxiety correlate with low birth weight,^7^^–^^15^ and difficulties in offspring including emotional and behavioural problems, cognitive impairment and psychopathology.^16^^–^^20^ Understanding the causes and prevalence of mental health problems in pregnancy and the early postpartum period is important for clinical management.

In 2015, we initiated a Medical Research Council funded study to examine antenatal and postpartum maternal mood disorders in a local Welsh population focusing on women undergoing an elective caesarean section (ELCS). ELCS was chosen to maximise the efficient collection of biological samples, which will be described elsewhere. The proportion of hospital deliveries by caesarean section in Wales rose from 24% in 2002–2003 to 26% in 2015–2016 with 11.8% being elective (http://gov.wales/statistics-and-research/maternity-statistics) highlighting the increasing importance of studying this population. Few studies report on the longer-term progression of depression and anxiety symptoms in new mothers after a planned surgical delivery that may differ from other modes if requested, for example, because of fear of childbirth or previous experience of trauma.^2^ Moreover, during pregnancy and labour dramatic changes occur in the brain resulting in physiological and behavioural adaptations that enable women to cope with motherhood.^21^ Mothers delivering by ELCS do not undergo the physiological process of labour that may have longer-term consequences. In this first report on the Grown in Wales (GiW) study, we determined antenatal and postpartum prevalence and progression of anxiety and depression symptoms reported by women undergoing ELCS using validated questionnaires, and examined the relevance of risk factors variously linked to antenatal depression and anxiety including a previous history of mental illness, alcohol, smoking, maternal age, parity, maternal body mass index (BMI), education level and income.^2^^,^^5^^,^^22^^–^^27^ We included fetal gender as a potential predictor as some studies report that women who give birth to boys are more likely to experience postpartum depression.^28^^,^^29^

## Method

### Study design, participants and ethics

Women were recruited at their pre-operative assessment 1 day prior to an ELCS between the ages of 18 to 45 with a singleton term pregnancy excluding fetal anomalies and infectious diseases by two research midwives between 1 September 2015 and 31 November 2016, recording indication for delivery mode. In total, 355 women agreed to participate. Of these, 317 women reported White ethnicity, of which 1 withdrew and 8 failed to complete the questionnaire (supplementary figure 1 available at https://doi.org/10.1192/bjo.2018.48) approval for the study was obtained via the Wales Research Ethics Committee REC reference 15/WA/0004.

### Measures

The questionnaire (A1) completed at recruitment consisted of two assessments of perceived mood symptoms. Depression symptoms were assessed using the Edinburgh Postnatal Depression Scale (EPDS)^30^ and trait anxiety was assessed using a subscale from the Spielberger State-Trait Anxiety Inventory (STAI) test (form Y-2).^31^^,^^32^ Questions on ethnicity (both parents), place of birth, age, weight (pre-pregnancy and current), income, mental health history and lifestyle were also included. Participants provided a sample of saliva in the morning at least 30 min after their last meal. Samples were kept at −80°C until cortisol concentration in μg/dL was determined in duplicate repeats by the Human Tissue Authority licensed Salimetrics at Anglia Ruskin University. Participants repeated the EPDS and STAI questionnaires within 7 days of delivery (P1) and then 10 weeks (P2) and 1 year (Y1) postpartum. Women reporting high anxiety/depression scores postpartum were contacted by R.P. or N.A.S. to ensure access to appropriate help.

### Additional data collected

Antenatal data were recorded from National Health Service (NHS) maternity records including mental health history, medication taken during pregnancy and complications during the pregnancy for comparison with questionnaire data. Welsh Index of Multiple Deprivation (WIMD) 2014 scores were calculated from anonymised postcodes (http://wimd.wales.gov.uk). Delivery information, fetal and placental biometry were recorded, and biological samples collected.

### Data analysis

Analyses were conducted using IBM SPSS Statistics for Macintosh, Version 23.0. Univariate analyses was performed to assess the relationships between maternal antenatal depression and maternal factors. Antenatal depression data was non-normally distributed and independent samples Mann–Whitney *U*-test, χ^2^-test, Friedman test, Wilcoxon signed ranks test, independent samples Kruskal–Wallis test and Spearman's rho correlations were used to analyse data. In tables results are displayed as *n* (%) for categorical variables and median (interquartile range) for continuous variables. Binary logistic regression was used to identify predictors of A1 EPDS scores ≥13. All data presented as bar charts are shown as mean (with standard error of the mean). *P*≤0.05 were considered statistically significant.

## Results

### GiW cohort

The majority (91%; *n* = 308) of eligible GiW participants reported White ethnicity with 74% reporting Wales as their birth place (supplementary Table 1). To avoid potential confounders introduced by heterogeneous sampling in smaller cohort studies,^33^^,^^34^ we focused on this group. One in four of these participants reported a mental health history on our questionnaires. Examination of NHS maternity records revealed an additional 12 participants with a history not recorded on their questionnaire (26% versus 30%, supplementary Table 2), which may reflect the concerns some women have about reporting their mental health history. Similarly, fewer women (6.2%) reported using antidepressants in pregnancy in our questionnaire compared with NHS maternity records on prescriptions (8.4%; supplementary Table 2), considerably higher than the prevalence reported in Wales between 2004 and 2010 (4.5%), already one of the highest rates in Europe.^35^

### Prevalence of maternal depression symptoms prior to delivery

EPDS scores of ≥13 predict an episode of clinical depression based on diagnostic criteria in the postpartum period.^36^^–^^38^ In total, 14.3% of women scored ≥13 on the EPDS questionnaire completed just prior to birth (A1; 37–42 weeks of pregnancy; Table 1). McCabe-Beane *et al.*^39^ further classified severity ranges in EPDS scores to identify no or minimal depression (EPDS 0–6), mild depression (EPDS 7–13), moderate depression (EPDS 14–19) and severe depression (>19).^19^^–^^30^ Using these severity ranges, during pregnancy 46% of participants had mild depression, 8% moderate depression and 3% severe depression.

**Table 1:** Summary of Grown in Wales prevalence data and data from similar studies

Population, condition and time point	% (95% CI)
*Wales (Cardiff), Current study 2018*	
Depression, EPDS ≥13	
37–42 weeks of pregnancy (*n* = 308)	14.3 (10.9–18.3)
4.5 days after birth (*n* = 163)	8.0 (4.2–12.5)
10 weeks after birth (*n* = 149)	8.7 (4.5–13.8)
1 year after birth (*n* = 113)	12.4 (6.8–18.4)
Anxiety, STAI ≥40	
37–42 weeks of pregnancy (*n* = 308)	27.3 (22.5–32.4)
4.5 days after birth (*n* = 166)	21.7 (15.8–28.0)
10 weeks after birth (*n* = 150)	25.3 (18.3–32.7)
1 year after birth (*n* = 114)	35.1 (26.3–44.2)
*England (Bristol), Heron et al. (2004)*^5^	
Depression, EPDS ≥13	
32 weeks of pregnancy (*n* = 8323)	13.1
6 weeks after birth (*n* = 8323)	8.9
Anxiety, CCEI ≥9	
32 weeks of pregnancy (*n* = 8323)	15.5
6 weeks after birth (*n* = 8323)	8.1
*England (South London), Howard (2018)*^3^	
Depression, EPDS and Structured Clinical Interview DSM-IV	
Within 3 weeks of first antenatal appointment (*n* = 545)	11
Anxiety, EPDS and Structured Clinical Interview DSM-IV	
Within 3 weeks of first antenatal appointment (*n* = 545)	15
*Sweden, Josefsson et al. (2001)*	
Depression, EPDS ≥10	
35–36 weeks of pregnancy (*n* = 1558)	17
3 days after birth (*n* = 1558)	18
6–8 weeks after birth (*n* = 1558)	13
*Australia (Melbourne), Leigh & Milgrom (2008)*	
Depression, BDI ≥16.5	
28–32 weeks of pregnancy (*n* = 278)	16.9
10–12 weeks after birth (*n* = 161)	11.2
Anxiety, BAI ≥16	
28–32 weeks of pregnancy (*n* = 278)	27.7
*Canada (Vancouver), Falah-Hassani et al. (2016)*	
Depression, EPDS ≥10	
1 week after birth (*n* = 522)	29.6
8 weeks after birth (*n* = 501)	20.4
Anxiety, STAI ≥40	
1 week after birth (*n* = 522)	20.1
8 weeks after birth (*n* = 501)	14.6

EPDS, Edinburgh Postnatal Depression Scale; STAI, State-Trait Anxiety Inventory; CCEI, Crown–Crisp Experiential Index; BDI, Beck Depression Inventory; BAI, Beck Anxiety Inventory.

### Comparison of characteristics of women scoring <13 with those scoring ≥13 on the EPDS scale

In a univariate analysis, women scoring ≥13 on the A1 EPDS questionnaire reported a lower level of education (*P* = 0.028) and lower family income (*P* = 0.029) consistent with a lower average WIMD score (supplementary Table 3). High scorers had a higher BMI at booking (27.5 versus 26.0, *P* = 0.012) and reported higher levels of smoking (*P* = 0.033) but not higher levels of drinking (*P* = 0.243), were more likely to have a mood disorder history (NHS maternity records; *P* < 0.001) and a higher A1 STAI score (*P* < 0.001). Some studies report that mothers with depression have higher antenatal cortisol levels whereas others report no association.^40^ We found no association between salivary cortisol provided the morning before the ELCS and EPDS score at A1 (*P* = 0.976; supplementary Table 3). Fetal gender ratio, gestational age, placental weight, birth weight, custom birth weight centile and head circumference was similar between those with high and low EPDS scores.

### Predictors of EPDS ≥13

Binary logistic regression was undertaken to identify variables that predict an A1 EPDS ≥13. Variables entered into multivariate model 1 (Table 2) were those significantly associated with A1 EPDS ≥13 in the univariate analysis (supplementary Table 3), as well as potential risk factors identified in the literature (alcohol, age, parity and fetal gender). Multicollinearity among independent variables was assessed and found within income and education. Consequently, income was excluded from the model. Model 1 was found to be significant with χ^2^(12) = 122.88, *P* < 0.001, and Nagelkerke *R*^2^ = 0.62, identifying significant predictors of A1 EPDS ≥13 to be A1 STAI total, fetal gender and education (left before GCSE).

**Table 2:** Binary logistic regression to establish predictors of A1 EPDS ≥13.

Predictor variables	OR	95% CI	*P*
*Multivariate model 1*			
Maternal Age	1.05	0.93–1.19	0.425
BMI	1.05	0.95–1.16	0.350
Smoked during pregnancy^a^	0.26	0.05–1.26	0.095
Alcohol during pregnancy^a^	1.41	0.50–3.97	0.517
Education			
Left before GCSE	12.38	1.26–121.16	0.031*
GCSE/O level and vocational	5.26	0.93–29.63	0.060
A levels	4.36	0.60–31.55	0.144
University	4.21	0.90–19.76	0.069
Postgraduate	Reference	Reference	Reference
Parity	0.40	0.10–1.66	0.206
Fetal gender	3.49	1.14–10.69	0.029*
Mental health history	2.71	0.94–7.80	0.065
A1 STAI total	1.37	1.23–1.52	<0.001*
*Multivariate model 2 (with non-significant predictors removed)*			
Smoked during pregnancy^a^	0.31	0.07–1.40	0.127
Education			
Left before GCSE	7.04	1.02–48.81	0.048*
GCSE/O level and Vocational	3.89	0.86–17.53	0.077
A levels	3.52	0.57–21.79	0.175
University	3.62	0.83–15.80	0.088
Postgraduate	Reference	Reference	Reference
Fetal gender	2.86	1.00–8.13	0.049*
Mental health history	3.15	1.17–8.51	0.024*
A1 STAI total	1.34	1.22–1.48	<0.001*

Results in bold are significant. A1, completed questionnaires at recruitment just prior to the birth; STAI, State-Trait Anxiety Inventory.

a. During at least one trimester of pregnancy.

* *P*<0.05.

A second binary logistic regression to produce model 2 (Table 2) was then undertaken, with non-significant variables in model 1 at *P* ≥0.1 removed. Model 2 was significant with χ^2^(8) = 120.02, *P* < 0.001, explaining 60.9% of the variation in A1 EDPS scores (Nagelkerke *R*^2^), only a slight decrease in fit from model 1. Model 2 identified significant predictors of A1 EPDS ≥13 to be A1 STAI total, mental health history, fetal gender and education (left before GCSE). A one-point increase in STAI total and a fetal gender of female compared with male increased the odds of A1 EPDS ≥13 by a factor of 1.34 and 2.86, respectively. Additionally, an education that ended before GCSE compared with postgraduate education, and a mental health history compared with no history increased the odds of having an A1 EPDS >13 by a factor of 7.04 and 3.15, respectively.

### Prevalence and progression of postpartum depressive symptoms

Mean completion of questionnaire P1 was 4.2 days postpartum (range 1–33, s.d. = 3.8), P2 was 69.1 days (range 40–112, s.d.=13.8) and Y1 was 405.8 days (range 353–561, s.d. = 45.3) (supplementary Fig. 1). There was no difference in P1 EPDS score (6.0 versus 6.0; *P* = 0.981) between participants completing questionnaires in hospital or at home. Prevalence of EPDS ≥13 at P1, P2 and Y1 was 8.4%, 8.6% and 12.4%, respectively (Table 1). Some studies report that women who give birth to boys are more likely to experience postpartum depression and anxiety.^28^^,^^29^ In the GiW cohort, postpartum EPDS and STAI scores were similar between mothers delivering boys and those delivering girls. However, salivary cortisol showed a relationship to gender with mothers delivering boys having significantly higher levels the morning before their ELCS (*P* = 0.004; supplementary Table S4).

**Fig. 1:**
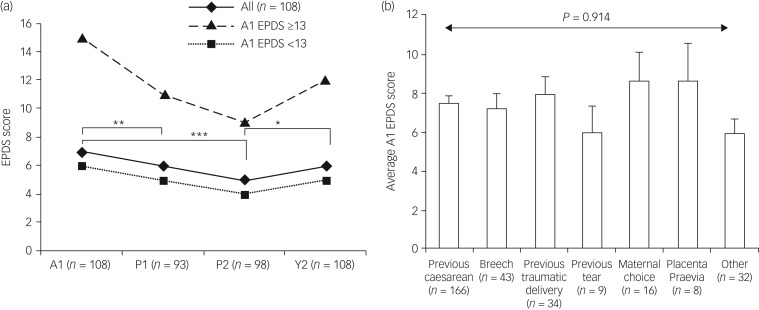
Progression of symptoms on the Edinburgh Postnatal Depression Scale (EPDS) and indications for elective caesarean section (ELCS).

A total of 108 participants (35%) completed questionnaires at A1, P1 and/or P2 and Y1, allowing the direct comparison of the progression of depressive symptoms (Fig. 1(a)). A Freidman test showed a significant difference in EPDS scores antenatally and postnatally (χ^2^(3) = 9.161, *P* = 0.027). *Post hoc* analysis using Wilcoxon signed-rank test with Bonferroni correction showed maternal EPDS scores to be significantly higher in late pregnancy compared with postnatal week 1 (7 versus 6; *Z* = −2.791, *P* = 0.005) and 10 weeks after delivery (7 versus 5; *Z* = −3.546, *P* < 0.001), similar to previous studies (Table 1). Postnatal Y1 scores were significantly higher than 10 weeks after delivery (5 versus 6; *Z* = −2.522, *P* = 0.012; Fig. 1).

(a) Progression of reported depression symptoms in participants who delivered by ELCS and completed questionnaires at recruitment just prior to the birth (A1), within 7 days of delivery (P1) and then 10 weeks (P2) and 1 year (Y1) postpartum. EPDS scores at P1 and P2 were significantly lower compared with late pregnancy EPDS scores for all participants. EPDS scores at postnatal Y1 was significantly higher than at week 10. (b) Reported depression symptoms in pregnancy according to indication for ELCS for 308 women who delivered by ELCS. Bar chart (mean with whiskers showing standard error of the mean) shows participants’ indication for ELCS section relative to antenatal depression scores as measured by the EPDS. ‘Previous caesarean’ covered elective and emergency procedures. Reason for ‘maternal choice’ was not recorded. ‘Other’ included prolapse, macrocephaly, severe endometriosis, failure to progress, gestational diabetes mellitus, maternal disorder (unspecified), high body mass index, bicornuate uterus, in vitro fertilisation/previous recurrent miscarriage, fetal growth restriction, previous neonatal death and large-for-gestational age. Depression scores were not significantly different for participants’ indication for section. *P < 0.05, **P < 0.01 and ***P < 0.005

All participants in this study were recruited from a cohort booked for an ELCS. In an independent samples Kruskal–Wallis test, there was no significant difference in late antenatal EPDS scores between participants with varying indications for ELCS (*P* = 0.914, Fig. 1(b)). Similarly, there was no significant difference in postnatal P1 (*P* = 0.947), postnatal P2 (i.e. week 10) (*P* = 0.424) or postnatal Y1 (*P* = 0.597) EPDS scores.

### Prevalence of anxiety symptoms

Trait anxiety was assessed using a subscale from the STAI test (form Y-2). Scores ≥40 indicate clinically significant symptoms of anxiety^41^ whereas >44 indicates major anxiety.^42^ There was no difference in STAI scores (30.0 versus 32.0; *P* = 0.573) between participants completing questionnaires in hospital or at home. Prevalence of STAI ≥40 at A1, P1, P2 and Y1 was 27.3%, 21.7%, 25.3% and 35.1%, respectively (Table 1). The prevalence of major anxiety symptoms (STAI >44) at A1, P1, P2 and Y1 was 12.0%, 14.5%, 17.3% and 17.9%, respectively.

### Comparison of characteristics of women scoring <40 with those scoring ≥40 on the STAI scale

In contrast to EPDS depression scores, a univariate analysis did not reveal any association between a STAI score ≥40 and education, family income, BMI or smoking. There was no association between salivary cortisol provided the morning before the ELCS and STAI score at A1 (*P* = 0.5; supplementary Table S5). Only mental health history (NHS maternity records; *P* < 0.001), type of mental health history (*P* = 0.009) and a higher A1 EPDS score (*P* < 0.001) were significantly associated.

### Progression of anxiety symptoms

**Fig. 2:**
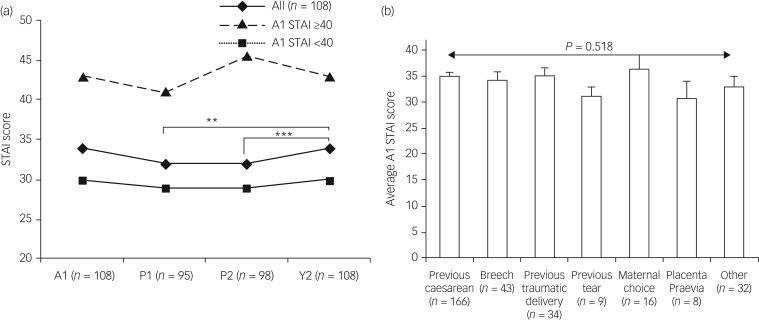
Progression of symptoms on the State-Trait Anxiety Inventory (STAI) and indications for elective caesarean section (ELCS).

Anxiety scores significantly differed between time points (χ^2^(3) = 10.047, *P* = 0.018; Fig. 2(a)). *Post hoc* analysis using Wilcoxon signed-rank test with Bonferroni correction revealed that this was not a consequence of decreased maternal anxiety scores after delivery as there was no difference between the antenatal score and postnatal scores either in week 1 (P1; *Z* = −1.678, *P* = 0.093) or in week 10 (P2; *Z* = −0.907, *P* = 0.364). This finding was in contrast to the majority of studies that report a decline in anxiety symptoms after delivery (Table 1). Furthermore, postnatal Y1 scores were significantly higher compared with postnatal P1 (34 versus 32; *Z* = −2.810, *P* = 0.005) and postnatal week 10 (P2) scores (34 versus 32; *Z* = −2.966, *P* = 0.003) indicating increasing anxiety symptoms. Reported anxiety symptoms did not differ by indications for ELCS either before delivery (independent samples Kruskal–Wallis test; *P* = 0.518) or after delivery at P1 (*P* = 0.975), P2 (*P* = 0.634) or Y1 (*P* = 0.611) (Fig. 2(b)) suggesting that prolonged anxiety was not a consequence of a specific indicator.

(a) Progression of reported anxiety symptoms in participants who delivered by ELCS and completed questionnaires at recruitment just prior to the birth (A1), within 7 days of delivery (P1) and then 10 weeks (P2) and 1 year (Y1) postpartum. For all participants, STAI scores did not significantly increase or decrease between antenatal A1 score and postnatal time points P1 or P2. STAI scores at postnatal Y1 was significantly higher than in week 1 and week 10. (b) Reported anxiety symptoms in pregnancy according to indication for ELCS section. Bar chart (mean with whiskers showing standard error of the mean) showing participants indication for ELCS section relative to antenatal anxiety scores as measured by the STAI. Anxiety scores were not significantly different for participants’ indication for section. ‘Previous caesarean’ covered elective and emergency procedures. Reason for ‘maternal choice’ was not recorded. ‘Other’ included prolapse, macrocephaly, severe endometriosis, failure to progress, gestational diabetes mellitus, maternal disorder (unspecified), high body mass index, bicornuate uterus, in vitro fertilisation/previous recurrent miscarriage, fetal growth restriction, previous neonatal death and large-for-gestational age. *P < 0.05, **P < 0.01 and ***P < 0.005

### Questionnaire return statistics

To examine the possibility that anxious women were more likely to return questionnaires, the characteristics of responders and non-responders were compared (supplementary Table S6). Non-responders were more likely to report a lower educational attainment (*P* = 0.001), lower household incomes (*P* < 0.001) and had a lower WIMD score (*P* < 0.001), were younger (*P* < 0.001) and more likely to smoke (*P* = 0.002) than responders but there were no differences in antenatal scores for depression, anxiety or mental health history.

Taken together, these data demonstrate that women who undergo an ELCS delivery experience prolonged anxiety lasting more than 1 year.

## Discussion

Our study findings on women in Wales delivering by ELCS mirrored the high prevalence of both depressive and anxiety symptoms in late pregnancy reported for other populations (Table 1). Whereas depressive symptoms decreased after delivery, symptoms of anxiety reported prenatally did not resolve with the safe delivery of a child. This raises the possibility that the physiology of a laboured pregnancy is important in ameliorating pregnancy-related anxiety, with implications for maternal welfare and the mother's growing relationship with her baby.

### Maternal depression decreases after delivery

In total, 14.3% of our mothers reported significant symptoms of depression prior to delivery and this number decreased to 8.4–8.6% after delivery, similar to other studies using the same EPDS questionnaire cut-offs. As with other studies, we found higher reported antenatal depression symptoms in pregnant women with lower family incomes, lower education qualifications and higher BMIs at their first antenatal booking.^2^ In the final model of the binary logistic regression in which non-significant predictors were removed (Table 2), low educational attainment, fetal gender, mental health history and A1 STAI score remained significant predictors of EPDS score. Female gender increased the odds of a higher EPDS score at A1 and mothers delivering girls had significantly lower salivary cortisol levels on the morning before their ELCS. Although we found no association between salivary cortisol and STAI score, more anxious mothers are already known to start the day with lower cortisol values than those with lower anxiety.^43^ To our knowledge this is the first report of an association between fetal gender and prenatal depression symptoms in a Western population.

### Maternal anxiety remains elevated after delivery

One in four GiW mothers reported significant symptoms of anxiety before their ELCS. In contrast to other studies, mothers with STAI ≥40 prenatally remained highly anxious throughout the 12 months of the study and the overall prevalence of women reporting anxiety symptoms did not significantly reduce after delivery with one in three women reporting significant symptoms 1 year after a safe delivery. This was not explained by anxious mothers being more likely to return questionnaires as there were no differences in antenatal STAI scores between responders and non-responders.

A study in Norway reported higher maternal distress before an ELCS compared with other modes of delivery and, although symptoms reduced somewhat 6 months after delivery, they were still higher than for other modes^44^ suggesting a link between this mode of delivery and anxiety. The majority of women in our study were undergoing an ELCS as a consequence of a prior caesarean delivery or a previous traumatic delivery. However, we did not observe a correlation between anxiety scores and indications for surgical delivery that might be anticipated if women's anxiety stemmed from a previous experience of trauma. STAI scores were similar across all indicators both before delivery and at all subsequent time points.

A key difference between ELCS and other modes of delivery is labour. Labour is initiated by a complex series of physiological and hormonal changes that women undertaking an ELCS do not experience. Dramatic changes occur in the brain resulting in physiological and behavioural adaptations that enable the prospective mother to cope with her new situation. A key hormone facilitating parturition is oxytocin, which has been shown in other mammals to play a critical role in the reduction of fear and anxiety postpartum.^21^ One possibility is that women remain anxious after ELCS because they do not undergo normal exposure to oxytocin, which could potentially be treated. Recognising and treating anxiety is important because untreated anxiety may impair maternal attachment and harm infant neurodevelopment.

### Study limitations

The prevalence of depression, risk factors and progression of symptoms from antenatal to postpartum time points in our GiW study compares well with previous studies on Western populations using the same EPDS questionnaire with similar time points and cut-offs (Table 1). However, previous studies report that anxiety symptoms resolve after delivery whereas they remained elevated in the women in this GiW study.

We had a higher than expected attrition rate after delivery. Our original study design was based on women remaining in hospital for 3–4 days after their ELCS allowing our research midwives to contact them directly but a change in hospital procedure meant many of our mothers went home only 1 day after delivery and had to return questionnaires by post, which may explain the lower response. Although there were demographic differences between responders and non-responders there were no differences in antenatal scores for depression, anxiety or mental health history, which reassures us that maternal mood was not a factor in the failure to return questionnaires.

A major limitation of this study is that we cannot say confidently that prolonged anxiety is a consequence of delivering by ELCS rather than a laboured birth because we did not recruit a cohort of women delivering by other modes to control for our specific population in Wales. There are a number of alternative explanations for our observation. Given the association between previous history of mood disorder and anxiety, the women delivering by ELCS may be more likely to have an ongoing anxiety disorder, although we would anticipate some evidence for this from the indications for surgical delivery. Welsh mothers may be more anxious than other populations or anxiety may be increasing in the general population. As in our study, women who have planned caesareans are more likely to be older than women delivering by other methods^45^ and older mothers are known to have significantly higher rates of depression than younger mothers^46^^,^^47^ that could also contribute to increased anxiety.

A larger population study comparing different modes of delivery within the same population will provide important context for our observation. Nonetheless, it is a major concern that one in three women report significant symptoms of anxiety over a 12-month period when they are caring for their infants.

A further weakness of our study is the lack of population diversity. In total, 83.7% of the population in the Cardiff local authority area (Stats for Wales 2017) and 84.7% of residents in Cardiff (2011 census data) were recorded as White. In our study, 91% of our eligible participant were White, which may be explained because we only recruited English-speaking participants. It is now critically important to ask whether other ethnicities experience prolonged anxiety after ELCS.

A third weakness of the study was the use of questionnaires that are inherently subjective. To counteract misreporting, we compared questionnaire responses with data recorded within NHS maternity records. More mothers reported smoking and drinking in the questionnaires whereas fewer accurately reported their mood history and antidepressant drug history. We did not consult general practitioner records and it is possible that more information may be missed from maternity records because of participants being reluctant to disclose as a result of concerns around stigmatisation or midwives not being appropriately trained to ensure they ask the questions correctly, factors that must be considered for all studies of this type.

In summary, a major concern that we identified in this study was the high prevalence of reported anxiety symptoms in women undergoing ELCS that do not resolve after delivery. One in four women before delivery and one in three women 1-year postpartum reported concerning symptoms indicating a failure of anxiety symptoms to resolve despite the safe delivery of a child. This is in contrast to previous reports on natural deliveries and emergency caesareans where the prevalence of reported anxiety symptoms reduces after birth.^48^ Future work should address how we support these high-risk women postpartum to attenuate their symptoms improving longer-term outcomes for them and their children.

## Supplementary material

For supplementary material accompanying this paper visit http://dx.doi.org/10.1192/bjo.2018.48. click here to view supplementary material
