# Supplementary material for: Persistence of anxiety symptoms after elective caesarean delivery
Source: BJPsych Open. 2018 Aug 17;4(5):354–60. doi: 10.1192/bjo.2018.48 (PMC6127961; doi:10.1192/bjo.2018.48)
Supplement: Supplementary file 1 [file S2056472418000480sup001.docx]

**Supplementary Table 1.** Demographic and characteristics of the GIW participants

| **Demographic** | **GIW** |
| --- | --- |
| ***Maternal characteristics*** | **(*n* = 308)** |
| Maternal Country of Birth  *Wales*  *England*  *Scotland*  *Ireland*  *Other*  *Not recorded* | 227 (74)  58 (19)  2 (1)  1 (<1)  18 (6)  2 (1) |
| Maternal Education  *Left before GCSE*  *GCSE/O level*  *A levels*  *University*  *Postgraduate*  *Vocational*  *Not recorded* | 19 (6)  58 (19)  38 (12)  92 (30)  75 (24)  17 (6)  9 (3) |
| Family Income  *<18,000*  *18 - 25,000*  *25 - 43,000*  *> 43,000*  *Do not wish to say*  *Not recorded* | 27 (9)  28 (9)  61 (20)  150 (49)  38 (12)  4 (1) |
| WIMD Score | 1212.0 (1253.0) |
| Maternal Age | 33.0 (6.0) |
| Parity  *Nulliparous*  *Multiparous* | 64 (21)  244 (79) |
| Weight Gained (kg) | 11.4 (6.3) |
| Maternal BMI | 26.2 (7.2) |
| BMI Category  *Underweight*  *Healthy*  *Overweight*  *Obese*  *Not recorded* | 3 (1)  119 (39)  106 (34)  79 (26)  1 (<1) |
| GDM Diagnosis  *No*  *Yes*  *Not recorded* | 289 (94)  15 (5)  4 (1) |
| Smoked Daily During Pregnancy  *No*  *Yes*  *Not recorded* | 285 (93)  21 (7)  2 (1) |
| Alcohol Weekly During Pregnancy  *No*  *Yes*  *Not recorded* | 278 (90)  22 (7)  8 (3) |
| Mood Disorder History^a^  *No*  *Yes*  *Not recorded* | 212 (69)  92 (30)  4 (1) |
| Type of Mood Disorder^b^  *Postnatal depression*  *Depression*  *Anxiety and depression*  *Anxiety*  *Other*^c^  *Not recorded* | 20 (22)  22 (24)  10 (11)  7 (8)  15 (16)  18 (20) |
| Medication for Mood Disorder^b^  *No*  *Yes* | 282 (92)  26 (8) |
| Type of Mood Disorder Medication^b^  *SSRI*  *Antidepressant (other)*  *Anti-anxiety*  *Other*  *Not recorded* | 22 (7)  1 (<1)  1 (<1)  2 (1)  282 (92) |
| Cortisol (μl/dL) | 0.30 (0.12) |
| A1 EPDS Total | 7.0 (6.0) |
| A1 STAI Total | 34.0 (12.0) |
| P1 EPDS Total | 6.0 (6.0) |
| P1 STAI Total | 32.0 (12.5) |
| P2 EPDS Total | 4.5 (7.0) |
| P2 STAI Total | 32.0 (13.5) |
| Y1 EPDS Total | 6.0 (6.0) |
| Y1 STAI Total | 33.0 (14.0) |
| ***Infant characteristics*** |  |
| Fetal Sex  *Female*  *Male*  *Not recorded* | 168 (55)  137 (45)  3 (1) |
| Gestational Age (weeks) | 39.0 (0.0) |
| Placental Weight (g) | 655.0 (185.5) |
| Birth Weight (g) | 3500.0 (645.0) |
| Custom Birth Weight Centile | 54.8 (49.7) |
| Head Circumference (cm) | 35.5 (2.0) |

WIMD: Welsh Index of Multiple Deprivation; BMI: body mass index; GDM: gestational diabetes mellitus; SSRI: selective serotonin reuptake inhibitor; EPDS: Edinburgh Postnatal Depression Scale; STAI: Stait-Trait Anxiety Inventory.

Results are displayed as (%) or median (IQR).

a. Mood disorder and medication history taken from medical notes.

b. Includes comorbid conditions such as ‘depression and eating disorder’, and ‘bipolar and depression’.

**Supplementary Table 2.** Comparison of data on GiW participants related to mood history, drinking and smoking data obtained from GiW A1 questionnaire versus data from medical notes.

| **Information** | **Questionnaire**  ***n* = 308)** | **Medical Notes**  **(*n* = 308)** |
| --- | --- | --- |
| Smoking during first trimester  *No*  *Yes*  *Not recorded* | 274 (89%)  33 (11%)  1 (<1%) | 281 (91%)  26 (8%)  1 (<1%) |
| Alcohol (any quantity) during first trimester  *No*  *Yes*  *Not recorded* | 230 (75%)  76 (25%)  2 (1%) | 293 (95%)  14 (5%)  1 (<1%) |
| Alcohol weekly during first trimester  *No*  *Yes*  *Not recorded* | 288 (94%)  18 (6%)  2 (1%) | 293 (95%)  14 (5%)  1 (<1%) |
| Mental health history  *No*  *Yes*  *Do not wish to say*  *Not recorded* | 225 (73%)  80 (26%)  2 (1%)  1 (<1%) | 212 (69%)  92 (30%)  0 (0%)  4 (1%) |
| Mood disorder medication  No  Yes  Not recorded | 286 (93%)  19 (6%)  3 (1%) | 281 (91%)  26 (8%)  0 (0%) |
| Medication type  SSRI  Antidepressants (other)  Anti-anxiety  Other | 15 (79%)  1 (5%)  0 (0%)  3 (16%) | 22 (85%)  1 (4%)  1 (4%)  2 (8%) |

SSRI: Selective serotonin uptake inhibitor.

**Supplementary Table 3.** Comparison of demographics between GIW participants (*n* = 308) who scored <13 and those scoring ≥13 on the EPDS questionnaire.

| Demographic | A1 EPDS <13 | A1 EPDS ≥13 | *P* value |
| --- | --- | --- | --- |
| *Maternal characteristics* | ***n* = 264** | ***n* = 44** |  |
| Education  *Left before GCSE*  *GCSE/O level*  *A levels*  *University*  *Postgraduate*  *Vocational*  *Not recorded* | 14 (5%)  45 (17%)  29 (11%)  80 (30%)  71 (27%)  16 (6%)  9 (3%) | 5 (11%)  13 (30%)  9 (21%)  12 (27%)  4 (9%)  1 (2%)  0 (0%) | **0.028** |
| Family Income  *<18,000*  *18 – 25,000*  *25 – 43,000*  *> 43,000*  *Do not wish to say*  *Not recorded* | 18 (7%)  23 (9%)  53 (20%)  135 (51%)  32 (12%)  3 (1%) | 9 (21%)  5 (11%)  8 (18%)  15 (34%)  6 (14%)  1 (2%) | **0.029** |
| WIMD Score | 1270.0 (1215.8) | 961.0 (1328.5) | 0.064 |
| Maternal Age | 33.0 (6.0) | 32.0 (7.0) | 0.384 |
| Parity  *Nulliparous*  *Multiparous* | 55 (21%)  209 (79%) | 9 (21%)  35 (80%) | 0.954 |
| Weight Gain (kg) | 11.4 (6.2) | 10.8 (7.7) | 0.349 |
| Maternal BMI | 26.0 (7.0) | 27.5 (8.5) | **0.012** |
| BMI Category  *Underweight*  *Healthy*  *Overweight*  *Obese*  *Not recorded* | 3 (1%)  108 (41%)  91 (35%)  62 (24%)  0 (0%) | 0 (0%)  11 (25%)  15 (34%)  17 (39%)  1 (2%) | 0.092 |
| GDM Diagnosis  *No*  *Yes*  *Not recorded* | 248 (94%)  14 (5%)  2 (1%) | 41 (93%)  1 (2%)  2 (5%) | 0.411 |
| Smoked During Pregnancy^a^  *No*  *Yes*  *Not recorded* | 237 (90%)  25 (10%)  2 (1%) | 35 (80%)  9 (21%)  0 (0%) | **0.033** |
| Weekly Alcohol Consumption During Pregnancy^b^  *No*  *Yes*  *Not recorded* | 240 (91%)  17 (6%)  7 (3%) | 38 (86%)  5 (11%)  1 (2%) | 0.243 |
| Mood Disorder History^c^  *No*  *Yes*  *Not recorded* | 199 (75%)  62 (24%)  3 (1%) | 13 (30%)  30 (68%)  1 (2%) | **0.001** |
| A1 STAI Total | 32.0 (10.8) | 46.0 (9.8) | **0.001** |
| Cortisol (μl/dL) | 0.30 (0.11) | 0.28 (0.17) | 0.976 |
| *Infant characteristics* | ***n* = 264** | ***n* = 44** |  |
| Fetal Sex  *Female*  *Male*  *Not recorded* | 143 (54%)  119 (45%)  2 (1%) | 25 (57%)  18 (41%)  1 (2%) | 0.664 |
| Gestational Age (weeks) | 39.0 (0.0) | 39.0 (0.0) | 0.575 |
| Placental weight (g) | 652.0 (183.0) | 660.5 (208.5) | 0.837 |
| Birth weight (g) | 3510.0 (662.5) | 3430.0 (570.0) | 0.819 |
| Custom birth weight centile | 58.1 (51.7) | 51.1 (41.9) | 0.169 |
| Head circumference (cm) | 35.5 (2.0) | 35.3 (2.0) | 0.755 |

WIMD: Welsh Index of Multiple Deprivation; BMI, body mass index; GDM: gestational diabetes mellitus; SSRI: selective serotonin reuptake inhibitor.

Results are displayed as (%) or median (IQR).

a. Smoked during at least one trimester of pregnancy.

b. Consumed alcohol at least once per week during at least one trimester or pregnancy.

c. Mood disorder and medication history taken from medical notes.

**Supplementary Table 4. Comparison of participants mental health characteristics between mothers giving birth to boys and those giving birth to girls.**

| **Mental health variable** | **Males (n = 137)** | **Females (n = 168)** | ***P* value** |
| --- | --- | --- | --- |
| A1 EPDS Score | 7.0 (5.0) | 7.0 (6.0) | 0.361 |
| P1 EPDS Score | 6.0 (7.5) | 6.0 (6.0) | 0.988 |
| P2 EPDS Score | 4.0 (7.0) | 5.0 (5.8) | 0.721 |
| A1 EPDS ≥ 13  *No*  *Yes* | 119 (87)  18 (13) | 143 (85)  25 (15) | 0.664 |
| A1 EPDS Severity  None/minimal  Mild  Moderate  Severe | 59 (43)  62 (45)  10 (7)  6 (4) | 76 (45)  76 (45)  14 (8)  2 (1) | 0.375 |
| P1 EPDS ≥ 13  *No*  *Yes*  *Not recorded* | 72 (53)  5 (4)  60 (44) | 80 (48)  9 (5)  79 (47) | 0.403 |
| P2 EPDS ≥ 13  *No*  *Yes*  *Not recorded* | 61 (45)  7 (5)  69 (50) | 78 (46)  6 (4)  84 (50) | 0.490 |
| A1 STAI Score | 34.0 (13.00) | 33 (11.8) | 0.282 |
| P1 STAI Score | 34.0 (13.5) | 31.0 (11.0) | 0.320 |
| P2 STAI Score | 31.0 (13.0) | 33.0 (13.0) | 0.291 |
| P2 STAI Score | 31.0 (13.0) | 33.0 (13.0) | 0.291 |
| A1 STAI ≥ 40  *No*  *Yes* | 97 (71)  40 (29) | 126 (75)  42 (25) | 0.411 |
| P1 STAI ≥ 40  *No*  *Yes*  *Not recorded* | 59 (43)  19 (14)  59 (43) | 73 (44)  18 (11)  77 (46) | 0.473 |
| P2 STAI ≥ 40  *No*  *Yes*  *Not recorded* | 52 (38)  16 (12)  69 (50) | 63 (38)  22 (13)  83 (49) | 0.738 |
| Y1 STAI ≥ 40  *No*  *Yes*  *Not recorded* | 52 (38)  16 (12)  69 (50) | 63 (38)  22 (13)  83 (49) | 0.738 |
| Cortisol (µg/dL) | 0.31 (0.14) | 0.28 (0.12) | **0.004** |

**Supplementary Table 5. Comparison of demographics between GIW participants (n = 308) who scored < 40 and those scoring ≥ 40 on the STAI questionnaire.**

| Demographic | STAI <40 | STAI ≥40 | *P* value |
| --- | --- | --- | --- |
| *Maternal characteristics* | **n = 224** | **n = 84** |  |
| Education  *Left before GCSE*  *GCSE or vocational training*  *A levels*  *University*  *Postgraduate*  *Not recorded* | 12 (5)  55 (25)  25 (11)  68 (30)  57 (25)  7 (3) | 7 (8)  20 (24)  13 (16)  24 (29)  18 (21)  2 (2) | 0.686 |
| Family Income  *<18,000*  *18 – 25,000*  *25 – 43,000*  *> 43,000*  *Do not wish to say*  *Not recorded* | 15 (7)  20 (9)  41 (18)  117 (52)  28 (13)  3 (1) | 12 (14)  8 (10)  20 (24)  33 (39)  10 (12)  1 (2) | 0.131 |
| WIMD Score | 1316.0 (1230.0) | 903.0 (1240.5) | 0.072 |
| Maternal Age | 33.0 (6.0) | 33.0 (6.0) | 0.880 |
| Parity  *Nulliparous*  *Multiparous* | 51 (23)  173 (77) | 13 (16)  71 (85) | 0.160 |
| Weight Gain (kg) | 11.4 (6.3) | 11.4 (6.9) | 0.282 |
| Maternal BMI | 26.1 (7.1) | 26.4 (7.9) | 0.159 |
| BMI Category  *Underweight*  *Healthy*  *Overweight*  *Obese*  *Not recorded* | 3 (1)  90 (40)  78 (35)  53 (24)  0 (0) | 0 (0)  29 (35)  28 (33)  26 (31)  1 (2) | 0.400 |
| GDM Diagnosis  *No*  *Yes*  *Not recorded* | 210 (94)  11 (5)  3 (1) | 79 (94)  4 (5)  1 (1) | 0.955 |
| Smoked During Pregnancy^a^  *No*  *Yes*  *Not recorded* | 201 (90)  21 (9)  2 (1) | 71 (85)  13 (16)  0 (0) | 0.170 |
| Smoking daily during pregnancy  *No*  *Yes*  *Not recorded* | 208 (93)  14 (6)  2 (1) | 77 (92)  7 (8)  0 (0) | 0.531 |
| Weekly Alcohol Consumption During Pregnancy^b^  *No*  *Yes*  *Not recorded* | 202 (90)  15 (7)  7 (3) | 76 (91)  7 (8)  1 (1) | 0.651 |
| Mood Disorder History^c^  *No*  *Yes*  *Not recorded* | 176 (79)  46 (21)  2 (1) | 36 (43)  46 (55)  2 (2) | **< 0.001** |
| Type of Mood Disorder^c^  *Depression*  *Postnatal depression*  *Anxiety and depression*  *Anxiety*  *Other*^d^  *None*  *Not recorded* | 16 (7)  9 (4)  2 (1)  4 (2)  3 (1)  176 (79)  12 (5) | 6 (7)  11 (13)  8 (10)  3 (4)  12 (14)  36 (43)  6 (7) | **0.009** |
| A1 EPDS Total | 6.0 (5.0) | 12.0 (5.8) | **< 0.001** |
| Cortisol (μl/dL) | 0.30 (0.10) | 0.29 (0.14) | 0.500 |
| *Infant characteristics* |  |  |  |
| Fetal Sex  *Female*  *Male*  *Not recorded* | 126 (56%)  97 (43%)  1 (<1%) | 42 (50)  40 (48)  2 (2) | 0.411 |
| Gestational Age (weeks) | 39.0 (0.0) | 39.0 (0.0) | 0.451 |
| Placental weight (g) | 650.5 (185.3) | 663.0 (186.0) | 0.254 |
| Birth weight (g) | 3500.0 (670.0) | 3535.0 (632.5) | 0.378 |
| Custom birth weight centile | 54.1 (53.2) | 58.8 (48.5) | 0.552 |
| Head circumference (cm) | 35.5 (2.0) | 35.5 (2.3) | 0.835 |

**Supplementary Table 6. Comparison of GiW questionnaire responders and non-responders.**

Participants that delivered by ELCS, who returned at A1, P1 and/or P2 and Y1 vs. participants who only returned at A1. Excluded participants are those who returned at only A1 and Y1, and those who returned at A1 and P1 and/or P2 but not Y1.

| **Demographic** | **Returned at all time points**  **(n = 109)** | **Only returned at A1**  **(n = 100)** | ***p* value** |
| --- | --- | --- | --- |
| ***Maternal characteristics*** |  |  |  |
| Maternal highest education  *Left before GCSE*  *GCSE or vocational training*  *A level*  *Undergraduate*  *Postgraduate*  *Not recorded* | 0 (0)  17 (16)  13 (12)  38 (35)  38 (35)  3 (3) | 11 (11)  30 (30)  13 (13)  24 (24)  17 (17)  5 (5) | **< 0.001** |
| Household income  *< £18,000*  *£18 – 25,000*  *£25 – 43,000*  *> £43,000*  *Do not wish to say*  *Not recorded* | 2 (2)  6 (6)  19 (17)  71 (65)  10 (9)  1 (1) | 16 (16)  9 (9)  20 (20)  32 (32)  20 (20)  3 (3) | **< 0.001** |
| WIMD Score | 1487 (928.0) | 817 (1205.5) | **< 0.001** |
| Maternal age | 35 (6.5) | 31 (7.0) | **< 0.001** |
| Parity  *Nulliparous*  *Multiparous* | 27 (25)  82 (75) | 20 (20)  80 (80) | 0.409 |
| Indication for ELCS  *Previous Caesarean Section*  *Breech*  *Previous traumatic delivery*  *Previous tear*  *Maternal choice*  *Placenta Previa*  *Other* | 57 (52)  16 (15)  11 (10)  6 (6)  7 (6)  4 (4)  8 (7) | 58 (58)  12 (12)  13 (13)  2 (2)  3 (3)  2 (2)  10 (10) | 0.562 |
| Maternal BMI at booking | 26.3 (6.7) | 26.2 (7.6) | 0.770 |
| BMI Category  *Underweight*  *Healthy*  *Overweight*  *Obese* | 0 (0)  42 (39)  46 (42)  21 (19) | 1 (1)  37 (37)  36 (36)  26 (26) | 0.443 |
| Smoking daily during pregnancy  *No*  *Yes*  *Not recorded* | 108 (99)  1 (1)  0 (0) | 87 (87)  11 (11)  2 (2) | **0.002** |
| Alcohol consumption weekly  *No*  *Yes*  *Not recorded* | 100 (92)  6 (6)  3 (3) | 85 (85)  10 (10)  5 (5) | 0.203 |
| History of diagnosed mood disorder  *No*  *Yes*  *Not recorded* | 78 (72)  30 (28)  1 (1) | 66 (66)  32 (32)  2 (2) | 0.603 |
| Maternal EPDS score  *Antenatal* | 7 (6.5) | 7 (5.0) | 0.438 |
| Maternal STAI score  *Antenatal* | 34 (13.0) | 34 (11.5) | 0.871 |
| Cortisol (μl/dL) | 0.29 (0.13) | 0.29 (0.11) | 0.980 |
| ***Infant characteristics*** |  |  |  |
| Fetal Gender  *Female*  *Male*  *Not recorded* | 65 (60)  44 (40)  0 (0) | 54 (54)  44 (44)  2 (2) | 0.510 |
| Placental weight (g) | 683 (173.3) | 650 (174.8) | 0.228 |
| Birth weight (g) | 3550 (715.0) | 3475 (612.5) | 0.166 |
| Custom birth weight centile | 62.5 (49.0) | 54.7 (51.4) | 0.112 |
| Head circumference (cm) | 35.5 (2.1) | 35.0 (2.0) | 0.078 |

WIMD: Welsh Index of Multiple Deprivation; BMI: body mass index.
